# Datasets from an impact evaluation of a targeted prekindergarten program

**DOI:** 10.1016/j.dib.2019.104881

**Published:** 2019-11-29

**Authors:** Bianca Montrosse-Moorhead, Shaun M. Dougherty, Tamika P. La Salle, Jennie M. Weiner, Hannah M. Dostal

**Affiliations:** a Department of Educational Psychology, University of Connecticut, USA; b Department of Leadership, Policy, and Organizations, Vanderbilt University, USA; c Department of Educational Leadership, University of Connecticut, USA; d Department of Department of Curriculum and Instruction, University of Connecticut, USA

**Keywords:** Prekindergarten, Evaluation dataset

## Abstract

Given general trends in extant research on the impact of prekindergarten and that the structure and implementation of prekindergarten programs vary by state, researchers, educators, and policymakers are raising questions about what works, for whom, under what conditions, and the cost-benefit of such endeavors. Yet not all states have formally examined program impacts and few datasets have been expressly collected to evaluate effects. The current data article represents empirical examinations in the state of Connecticut on the comparability of treatment and control groups, tests of the robustness of impact estimates, and the psychometric properties of outcome measures. Stata code for replication purposes is included.

**Table:** Specifications Table

Subject area	Education
More specific subject area	Early childhood education
Type of data	Numeric, text, table, figure
How data was acquired	Restricted-use state data repository
Data format	Raw
Parameters for data collection	The data was collected from prekindergarten students enrolled in Connecticut's School Readiness Program during the 2014–2015 and 2015–2016 school year. This program is specifically targeted toward children from low-income families and communities.
Description of data collection	Datasets are available from Connecticut's P20 WIN data repository, and are supplied by two different state government agencies. The Office of Early Childhood Education maintains records on students who have enrolled in Connecticut's School Readiness Program, including enrollment dates, enrollment location, and student demographic information. The State Department of Education maintains records of students who enter public schools in Connecticut from Kindergarten through Grade 12, including enrollment dates, enrollment location, and student demographics information. These data are supplemented by *Woodcock Johnson IV Tests of Achievement* scores measuring reading, oral language, picture vocabulary and mathematical skills for each student.
Data source location	Connecticut, United States of America
Data accessibility	Raw restricted use data are available from Connecticut's P20 WIN data repository http://www.ct.edu/p20win/request-data
Related research article	B. Montrosse-Moorhead, S. M. Dougherty, T. P. La Salle, The overall and differential effects of a targeted prekindergarten program: Evidence from Connecticut, Early Childhood Research Quarterly, 48, 2019, 134–145 [1].

**Table:** 

**Value of the Data** • The datasets represent samples from the treatment group and control group students, which can be compared to reveal the overall impact of attending prekindergarten. • The datasets also contain demographic information on both treatment and control group students that can be used to explore variation in treatment effects. • Datasets are available in the form of raw numeric and text data that can be further processed by researchers using datasets from other states, their own data, or other datasets available in Connecticut's P20 WIN data repository.

## 1 Data

**Fig. 1:**
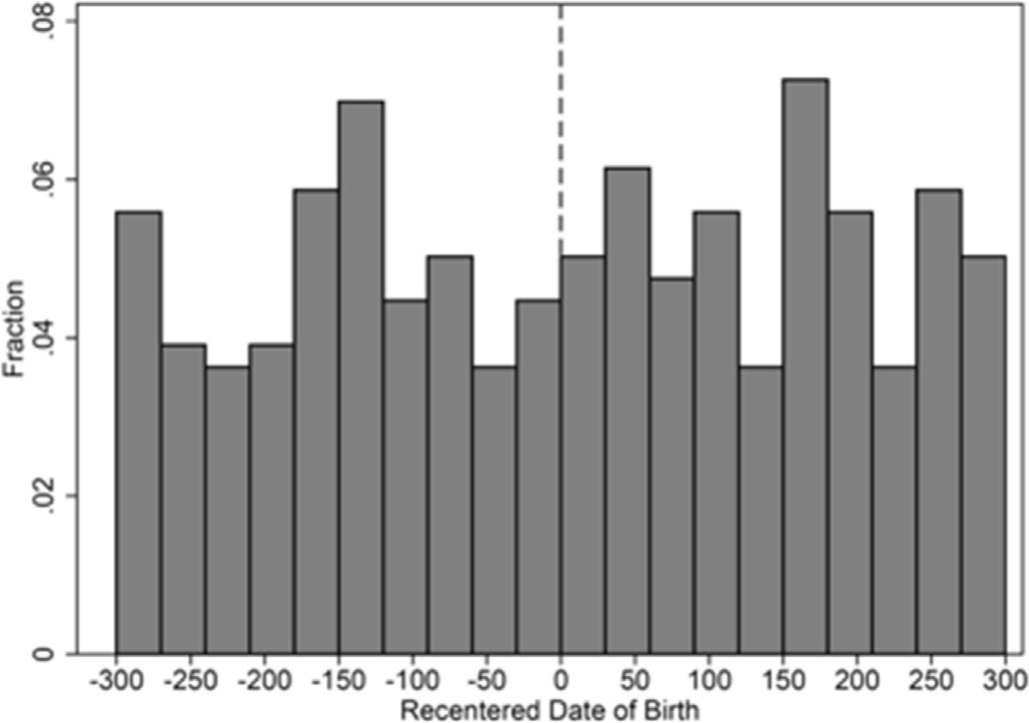
Distribution of the dates of birth around the January 1 enrollment cut-off.

The datasets contain raw numeric and text data available through Connecticut's P20 WIN data repository. A summary of data elements contained in the datasets used is presented in Table 1. Table 2 includes information on the comparability of treatment and comparison samples under different assumptions. Table 3, Table 4 include information on the robustness of impact estimates using linear, quadratic, and quintic models. Table 4 includes additional information on the robustness of impact estimates. Fig. 1 provides information on the smoothness and continuity of birthdates around the enrollment date cut-off.

**Table 1:** Data codebook for the datasets used by authors.

P20 WIN Index #	Source	Data Category	Data Element Name	Data Element Definition
17	SDE-SLDS	Student Reporting District	EntryDate	Date of student's entry into last facility in PSIS, as YYYYMMDD.
18	SDE-SLDS	Student Reporting District	ExitDate	Date of student's exit into last facility in PSIS, as YYYYMMDD.
19	SDE-SLDS	Student Reporting District	ReportingDistrictName	Name of district responsible for reporting the student's enrollment.
21	SDE-SLDS	Student	AmericanIndianOrAlaskaNative	Y-Yes if child is American Indian or Alaska Native. Otherwise N–No or NS-missing.
22	SDE-SLDS	Student	Asian	Y-Yes if child has origins in any of the original peoples of the Far East, Southeast Asia, or the Indian Subcontinent. Otherwise N–No or NS-missing.
23	SDE-SLDS	Student	BirthYear	Year portion of date of birth.
24	SDE-SLDS	Student	BlackOrAfricanAmerican	Y-Yes if child has origins in any of the black racial groups of Africa. Otherwise N–No or NS-missing.
26	SDE-SLDS	Student	FreeReducedLunchEligible	Indicator of student eligibility for federal free/reduced lunch program at any time.
27	SDE-SLDS	Student	Gender	Gender of the child. M = Male, F=Female.
30	SDE-SLDS	Student	HispanicOrLatino	Y-Yes if child's ethnicity is Cuban, Mexican, Puerto Rican, south or central American, or other Spanish culture or origin regardless of race. Otherwise N–No or NS-missing.
35	SDE-SLDS	Student	LastFacility1_SchoolName	Name of last facility.
44	SDE-SLDS	Student	NativeHawaiianOrOtherPacificIslander	Y-Yes if child has origins in any of the original peoples of Hawaii, Guam, Samoa, or other Pacific Islands. Otherwise N–No or NS-missing.
54	SDE-SLDS	Student	White	Y-Yes if child has origins in any of the original peoples of Europe, Middle East, or North Africa. Otherwise N–No or NS-missing.
55	SDE-SLDS	Student Assessment	Administered_FallOfYear	Year of fall of school year in which the test was administered.
56	SDE-SLDS	Student Assessment	Administered_SIFYear	Year of spring of school year in which the test was administered.
58	SDE-SLDS	Student Assessment	LevelScore	Student's performance level.
59	SDE-SLDS	Student Assessment	ScaleScore	Conversion of a student's raw score on a test to a common scale that allows for a numerical comparison between students.
60	SDE-SLDS	Student Assessment	TestType	Name of standardized assessment.
129	SDE-SLDS	Student	SASID	State assigned student identification number
393	OEC	Early Childhood	Enrollment ID	Enrollment ID is an ID number associated with SASID and specific funding type and dates for an enrollment record.
394	OEC	Early Childhood	FacilityCode	Facility code that is associated to the Early Care and Education Facility or school (7-digit code).
395	OEC	Early Childhood	FacilityName	Name of the Early Care and Education Facility or school.
397	OEC	Early Childhood	LastName	Child's legal first name that appears on the child's birth certificate or document indicating legal name change.
398	OEC	Early Childhood	FirstName	Child's legal last name that appears on the child's birth certificate or document indicating legal name change.
399	OEC	Early Childhood	MiddleName	Child's legal middle name that appears on the child's birth certificate or document indicating legal name change.
401	OEC	Early Childhood	DateOfBirth	Child's date of birth. Format MM/DD/YYYY.
402	OEC	Early Childhood	GenderCode	Gender of the child.
408	OEC	Early Childhood	IsHispanic	Y-Yes if child's ethnicity is Cuban, Mexican, Puerto Rican, south or central American, or other Spanish culture or origin regardless of race. Otherwise N–No or NS-missing.
409	OEC	Early Childhood	AmericanIndian	Y-Yes if child is American Indian or Alaska Native. Otherwise N–No or NS-missing.
410	OEC	Early Childhood	Asian	Y-Yes if child has origins in any of the original peoples of the Far East, Southeast Asia, or the Indian Subcontinent. Otherwise N–No or NS-missing.
411	OEC	Early Childhood	Black	Y-Yes if child has origins in any of the black racial groups of Africa. Otherwise N–No or NS-missing.
412	OEC	Early Childhood	Hawaiian	Y-Yes if child has origins in any of the original peoples of Hawaii, Guam, Samoa, or other Pacific Islands. Otherwise N–No or NS-missing.
413	OEC	Early Childhood	White	Y-Yes if child has origins in any of the original peoples of Europe, Middle East, or North Africa. Otherwise N–No or NS-missing.
430	OEC	Early Childhood	FacilityEntryDate	Date the child began attendance at the facility. Format MM/DD/YYYY.
431	OEC	Early Childhood	FacilityExitDate	Date the child ended attendance at the facility. Format MM/DD/YYYY.
434	OEC	Early Childhood	FundingType	Funding Type associated with the child's enrollment: 1 = School Readiness, competitive. 2 = School Readiness, priority. 3 = Child day care. 4 = Smart Start. 5 = PDG, federal. 6 = PDG, state quality enhancement. 7 = Head Start, state supplement. 8 = Head Start/Early Head Start. 9 = Private pay.
435	OEC	Early Childhood	SpaceType	Space type associated with the child's enrollment and funding: 1 = None. 2 = Full day, full year. 3 = Part day, part year. 4 = School day, school year. 5 = Infant/Toddler, full time. 6 = Infant/Toddler, wrap around. 7 = Preschool full time. 8 = Preschool wrap around. 9 = School age. 10 = Extended day. 11 = Extended year. 12 = State enrollment. 13 = Center-based full day. 14 = Center-based part day. 15 = No description available. 16 = Family child care. 17 = No description available. 18 = Full day expansion. 19 = Full day improved. 20 = School day expansion. 21 = School day expansion improved.

*Note*. SDE-SLDS is the state department of education's longitudinal data system. OEC is the dataset developed and maintained by the Office of Early Childhood Education.

**Table 2:** Covariate balance at birthday cut-off.

	Male	White	Black	Latino	Asian	Other	Low Income
IK bandwidth	0.066 (0.137)	0.062 (0.115)	0.075 (0.145)	−0.197 (0.141)	0.028 (0.027)	0.039 (0.058)	0.016 (0.121)
N	236	285	244	266	286	285	304
Bandwidth = 120 days	0.106 (0.194)	0.157 (0.167)	−0.022 (0.195)	−0.164 (0.198)	0.024 (0.021)	0.122 (0.093)	0.110 (0.161)
N	139	139	139	139	139	139	139
Bandwidth = 150 days	0.063 (0.168)	0.123 (0.145)	0.035 (0.170)	−0.181 (0.174)	0.023 (0.028)	0.098 (0.075)	0.041 (0.150)
N	178	178	178	178	178	178	178
Bandwidth = 180 days	0.079 (0.151)	0.098 (0.131)	0.071 (0.152)	−0.209 (0.157)	0.022 (0.029)	0.070 (0.067)	0.020 (0.139)
N	233	233	233	233	233	233	233
Bandwidth = 210 days	0.066 (0.138)	0.066 (0.121)	0.081 (0.137)	−0.201 (0.143)	0.025 (0.028)	0.047 (0.061)	0.026 (0.130)
N	259	259	259	259	259	259	259
Control Mean	0.529	0.235	0.382	0.441	0.000	0.029	0.735

*Note.* Heteroskedasticity robust standard errors clustered by birthdate are in parentheses. Each coefficient is the reduced form estimate of the relationship between being eligible for state-funded prek based on birthdate and the listed covariate. The coefficients shown are generated by local linear regression using a triangular kernel and specified bandwidth. The center of the specified bandwidth is the cut-off point. Also listed is the mean of the covariate for students just before the threshold for qualifying for prekindergarten. *p < .10, **p < .05, ***p < .01.

**Table 3:** Testing assumptions about functional form and bandwidth of impact estimates.

	Broad Math (SE)	Oral Language (SE)	Basic Reading (SE)	Picture Vocabulary (SE)
Linear, BW = IK	10.152*** (3.252)	5.459 (7.364)	10.828*** (3.079)	4.425 (4.906)
N	273	199	190	251
Quadratic, BW = IK	11.080*** (4.069)	12.954 (9.794)	13.125*** (4.221)	6.224 (7.588)
N	273	199	190	251
Quintic, BW = 365	10.302*** (5.217)	23.600** (11.041)	20.585*** (6.743)	15.858* (9.575)
N	425	425	425	418
Linear, BW = 180	10.601*** (3.370)	4.842 (5.750)	10.319*** (3.381)	4.696 (5.111)
N	219	219	219	215
Quadratic, BW = 180	10.878** (4.418)	12.113 (9.524)	13.432*** (4.014)	8.217 (7.929)
N	219	219	219	215
Linear, BW = 210	10.242*** (3.099)	4.028 (5.249)	10.352*** (3.152)	4.426 (4.629)
N	255	255	255	251
Quadratic, BW = 210	11.346*** (4.167)	10.144 (8.969)	12.568*** (3.853)	6.213 (7.586)
N	255	255	255	251

*Note.* *p < .10, **p < .05, ***p < .01.

**Table 4:** Test of robustness of impact estimates using different specifications with optimal bandwidth.

	Broad Math (SE)	Oral Language (SE)	Basic Reading (SE)	Picture Vocabulary (SE)
Baseline IK bandwidth model	7.198*** (2.865)	3.000 (6.005)	13.028*** (4.189)	1.216 (4.817)
N	299	236	113	262
Covariate-adjusted model	6.618*** (2.361)	1.349 (4.684)	12.658*** (4.410)	0.130 (3.892)
N	299	236	113	262
School clustered SE model	6.618*** (2.631)	1.349 (5.124)	12.658*** (4.027)	0.130 (4.075)
N	299	236	113	262
School FE model	12.133** (5.865)	15.674 (14.081)	8.524 (15.916)	9.038 (6.443)
N	299	236	113	262

*Note.* The coefficients shown were generated using OLS with the stated bandwidth and a triangular kernel. Models specified with covariates include indicator variables for gender, race/ethnicity, and eligibility for subsidized meals. *p < .10, **p < .05, ***p < .01.

## 2 Experimental design, materials, and methods

### 2.1 Ethical statements

This study was approved by the University of Connecticut's Institutional Review Board (Protocols H14–193 and H15-247), and carried out in accordance with a Memorandum of Agreement with the Connecticut State Department of Education and Office of Early Childhood and the Family Educational Rights and Privacy Act of 1974.

### 2.2 Collection of student educational record data

The Connecticut State Department of Education and Office of Early Childhood maintain monthly and yearly educational records for all students enrolled in public schools (prekindergarten through grade 12). This data includes, for each student, a unique identification number, name, date of birth, race/ethnicity, whether the student participates in the federal free and reduced priced lunch program, the school attended, and the school district in which the school is located. These data were provided to the research team through a Memorandum of Agreement.

More specifically, datasets are available through Connecticut's P20 WIN. To access datasets, researchers must apply to become an authorized representative. The request is then reviewed by members of the P20 WIN Data Governing Board, and if approved, a Memorandum of Agreement (MOA) specific to the data request is executed. Once executed, the authorized representative receives a matched dataset. All requestors are required to pay a fee. Under special circumstances the fee may be waived or reduced at the discretion of the Data Governing Board. The authorized representative and anyone on their team who has signed Personal Statements of Non-Disclosure is able to work with the data. All publicly shared information as a result of dataset use must be sufficiently aggregated to maintain confidentiality. A detailed articulation of how to access datasets is included in the P20 WIN Data Request Management Procedure manual available at http://www.ct.edu/files/pdfs/P20WIN-DataRequestProcedure-Final_01202015.pdf. The full set of restricted use datasets is available as a downloadable excel file at www.ct.edu/files/pdfs/p20win/P20WIN_Data_Dictionary.xlsx.

### 2.3 Collection and measurement of student outcome data

Student outcome data were collected by a team of 58 trained and certified assessors using the *Woodcock Johnson IV Tests of Achievement* (WJ-IV) and the *Peabody Picture Vocabulary Test* 4th *Edition* (PPVT-4). Data from these assessments were then scored and composite measures of student achievement were entered into a datafile by the research team. Four composite measures in total were used and entered into the datafile.

The Basic Reading composite included two subtests from the WJ-IV, specifically Word Attack and Letter-Word Identification. The Letter-Word Identification subtest measures pre-reading skills including letter and word recognition and identification skills. The Word Attack subtest measures phonics and decoding skills. The reliability statistics for individual tests range from 0.90 to 0.94 [2].

The Oral Language composite included two subtests (the Picture Vocabulary and Oral Comprehension) from the WJ-IV. The Picture Vocabulary subtest primarily assesses expressive vocabulary, though early items provide some information about receptive vocabulary skills. The Oral Comprehension subtest measures the ability to understand short, orally presented, passages. Subtest reliability statistics range from 0.82 to 0.88 [2].

The PPVT-4 measured participants' picture vocabulary, or students’ word knowledge ability. Spearman-Brown corrected, split-half, and alpha reliabilities consistently fall above 0.94, indicating solid internal consistency evidence [3].

The Broad Mathematical Skills composite consisted of three measures from the WJ-IV, specifically Applied Problems, Calculation, and Math Fluency subtests. The Applied Problems subtest was used to measure mathematics problem solving skills. The Calculation subtest measures students' ability to complete items ranging from writing numbers to performing numerical operations. And, the Math Fluency subtest measures students’ ability to solve problems containing numerical operations quickly. The reliability statistics for individual tests range from 0.96 to 0.97 [2].

### 2.4 Statistical analysis

All statistical analyses were conducted using Stata. For all student outcomes, the standard score with a mean of 100 with a standard deviation of 15 was used. A multilevel regression model with cluster standard errors was used because it does not force the same parametric assumptions about the distribution of the error terms at either the school or student level, or the correlation among them. The robustness of analysis decisions were evaluated through other specifications, including adding covariates, using an alternate clustering approach, and using fixed effects for each site. Unless otherwise indicated, p < 0.05 was considered statistically significant. The Stata code for analyzing data is included as a supplementary file.
